# Unexpected Diagnosis

**DOI:** 10.7759/cureus.5767

**Published:** 2019-09-26

**Authors:** Gauravpal S Gill, Rukma R Govindu, Ragai Fouda, Hussam M Ammar

**Affiliations:** 1 Internal Medicine, Medstar Washington Hospital Center, Washington DC, USA; 2 Internal Medicine, The University of Texas Health Science Center at Houston, Houston, USA; 3 Internal Medicine/cardiology, George Eliot Hospital Nhs Trust, Nuneaton , GBR; 4 Internal Medicine, Medstar Washington Hospital Center, Washington DC, USA

**Keywords:** pneumocystis pneumonia, aids, hiv, diagnostic momentum, diagnostic parsimony, occam’s razor, hicham’s dictum., limited english proficiency

## Abstract

A 74-year-old man presented to the ER with an eight-month history of shortness of breath, cough, anorexia, and weight loss. He had emigrated from sub-Saharan African to the USA, where he was diagnosed and treated for coronary artery disease, heart failure, and stroke; was hospitalized several times; and underwent hernia surgery. Despite the complex care that he received in the USA for many years, the diagnosis of AIDS was continually missed for years, and the patient was eventually diagnosed at the age of 74.

## Introduction

An estimated 1.1 million people in the USA live with HIV [1]. One in seven HIV-positive individuals is unaware of the infection, and 20% of individuals who are aware of their HIV diagnosis have not been linked to care [1-2]. Greater age is significantly associated with greater delays in diagnosis (median delay: 4.5 years in people aged ≥55 years vs. 2.4 years in people aged 13-24 years) [2]. Approximately 55% of adults in the USA have never been screened for HIV [3].This case report presents the journey of one of the 1.1 million patients living with HIV; this particular patient remained undiagnosed until the age of 74.

## Case presentation

**Figure 1:**
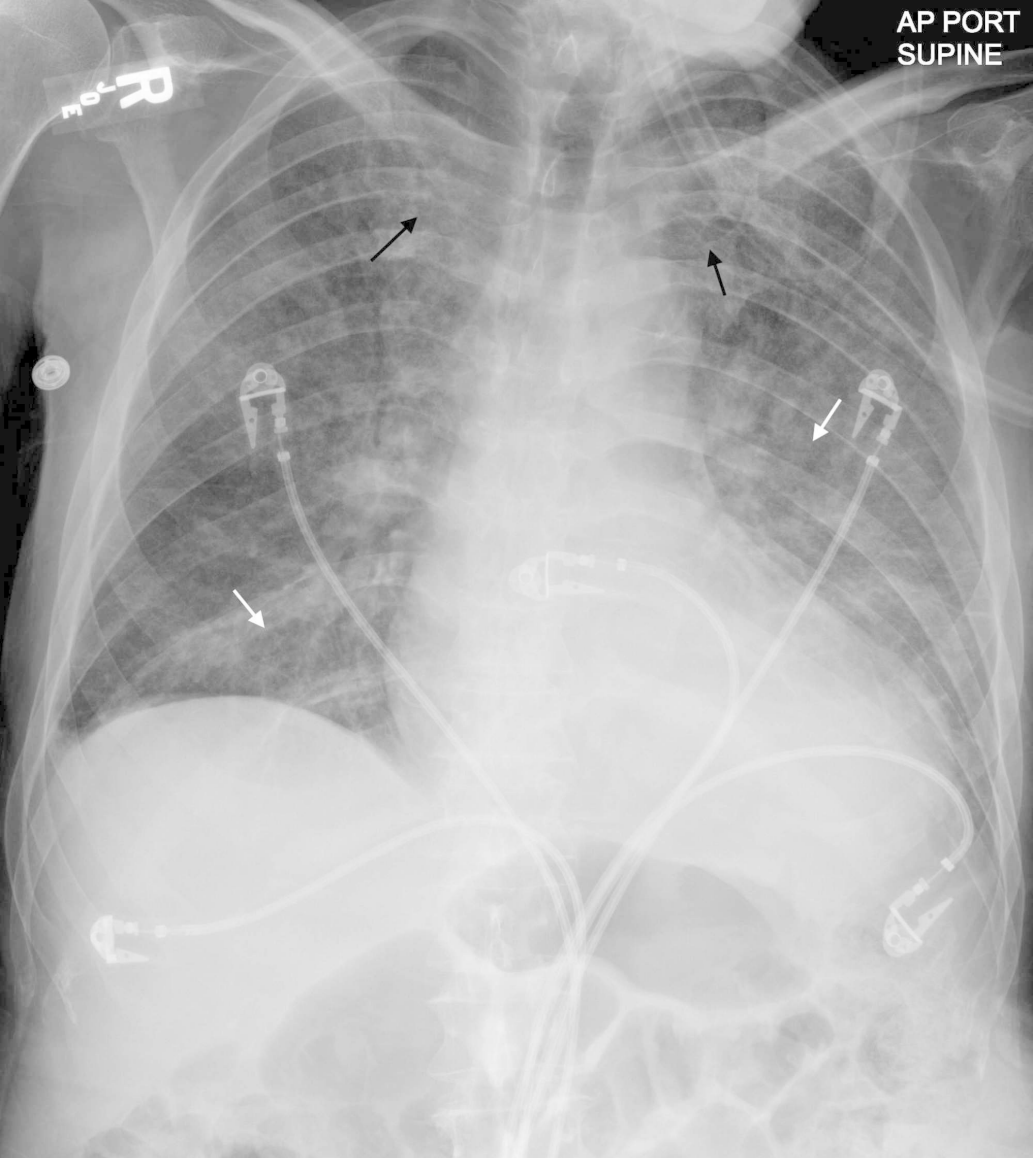
Portable anteroposterior chest radiography. Cephalization (black arrows), alveolar and reticular opacities (white arrows).

A 74-year-old man presented to the ER with an eight-month history of shortness of breath, coughing, anorexia, and weight loss without a fever. On examination, the patient was fully alert and oriented with a blood pressure of 80/60 mmHg, a regular pulse of 80 beats per minute, a respiratory rate of 18 breaths per minute, a temperature of 36.8°C, and a body mass index of 19 kg/m2. The jugular venous pulse pressure was elevated to 12 cm H2O, and bilateral fine basal crackles were audible on pulmonary auscultation. Cardiac examination revealed normal heart sounds and a holosystolic murmur with a grade of 3/6 that was best heard over the apex, with no gallop. He did not have lymphadenopathy or hepatosplenomegaly. The patient exhibited bitemporal wasting and diffuse wasting of the arm and leg muscles. His extremities were warm, and his peripheral pulses were palpable. Our patient had been previously diagnosed with congestive heart failure, atrial fibrillation, left ventricular aneurysm with left ventricular thrombus, and stroke, and two coronary stents had been placed a few years prior. He could walk only a few steps before feeling shortness of breath; he was fatigued most of the time, and he had lost approximately 7.7 kg in the previous year. His home medications included clopidogrel, apixaban, lisinopril, carvedilol, and furosemide. At the time of presentation, his serum albumin level was 1.6 g/dL, and his hemoglobin level was 11 g/dL. Chest radiography revealed cephalization and alveolar and reticular opacities (Figure 1).

The patient received 2 L of intravenous 0.9% sodium chloride in the ER. His blood pressure improved to 110/80 mmHg, and his creatinine level improved from 2.1 to 1.2 mg/dL. The patient, a retired businessman living with his family in the Southeast United States, was originally from sub-Saharan Africa and had immigrated to the USA 10 years prior. He had traveled to his home country in sub-Saharan Africa twice a year for the past 5 years. He had been receiving medical care in the USA and had undergone hernia surgery and percutaneous coronary intervention a few years previously. He had also been hospitalized a few times for pneumonia and diarrhea.

We interviewed the patient over the phone with the aid of an interpreter. He remembered that he had received a tuberculin skin test a decade prior, which produced a negative result. He did not recall any contact with patients with tuberculosis (TB). He reported experiencing intermittent watery, nonbloody diarrhea for the past six months, and he had used over-the-counter diphenoxylate, which resulted in some improvement in his symptoms.

The observed hypotension, elevated jugular venous pulse pressure, and fine basal crackles suggested a diagnosis of heart failure, but the patient’s warm extremities and normal mentation rendered a diagnosis of cardiogenic shock unlikely. We were careful not to fall into the traps of diagnostic momentum and assume that the clinical presentation was solely secondary to heart failure [4]. A few red flags emerged in this case. First, the patient was a foreign-born American citizen. The rate of TB is 13.4-fold higher among foreign-born persons in the USA than among US-born persons [5]. The language barrier prevented clear communication, which can sometimes lead physicians to incorrect diagnoses [6]. Although pneumonia and diarrhea diagnoses are not uncommon in a 74-year-old man, the patient’s multiple hospital admissions without a definitive diagnosis or successful treatment suggested a possible missed diagnosis. Sub-Saharan Africa, where this man traveled from and had lived for most of his life, has the highest prevalence of HIV infection in the world; this region is home to only 12% of the global population but accounts for 71% of the global burden of HIV infection [7]. The tuberculin test also has limitations. The test has a sensitivity of 71%-82% in patients with latent TB, but its sensitivity drops to only 43% in immunocompromised patients with HIV [8]. The findings on chest radiography could have been secondary to the known heart failure diagnosis of the patient, perhaps due to atypical pneumonia or fungal or viral infection. Pneumocystis pneumonia (PCP) can also cause a similar radiological pattern. He may also have had a combination of both heart failure and underlying infection.

The chest radiography findings did not rule out pulmonary TB; the incidence of radiographic findings that are not typical for primary or reactivated TB is 8%-29%, and even normal chest radiography results are observed in 1%-9.2% of patients with culture-proven pulmonary TB and in 22% of HIV-positive patients with pulmonary TB [9-10].

**Figure 2:**
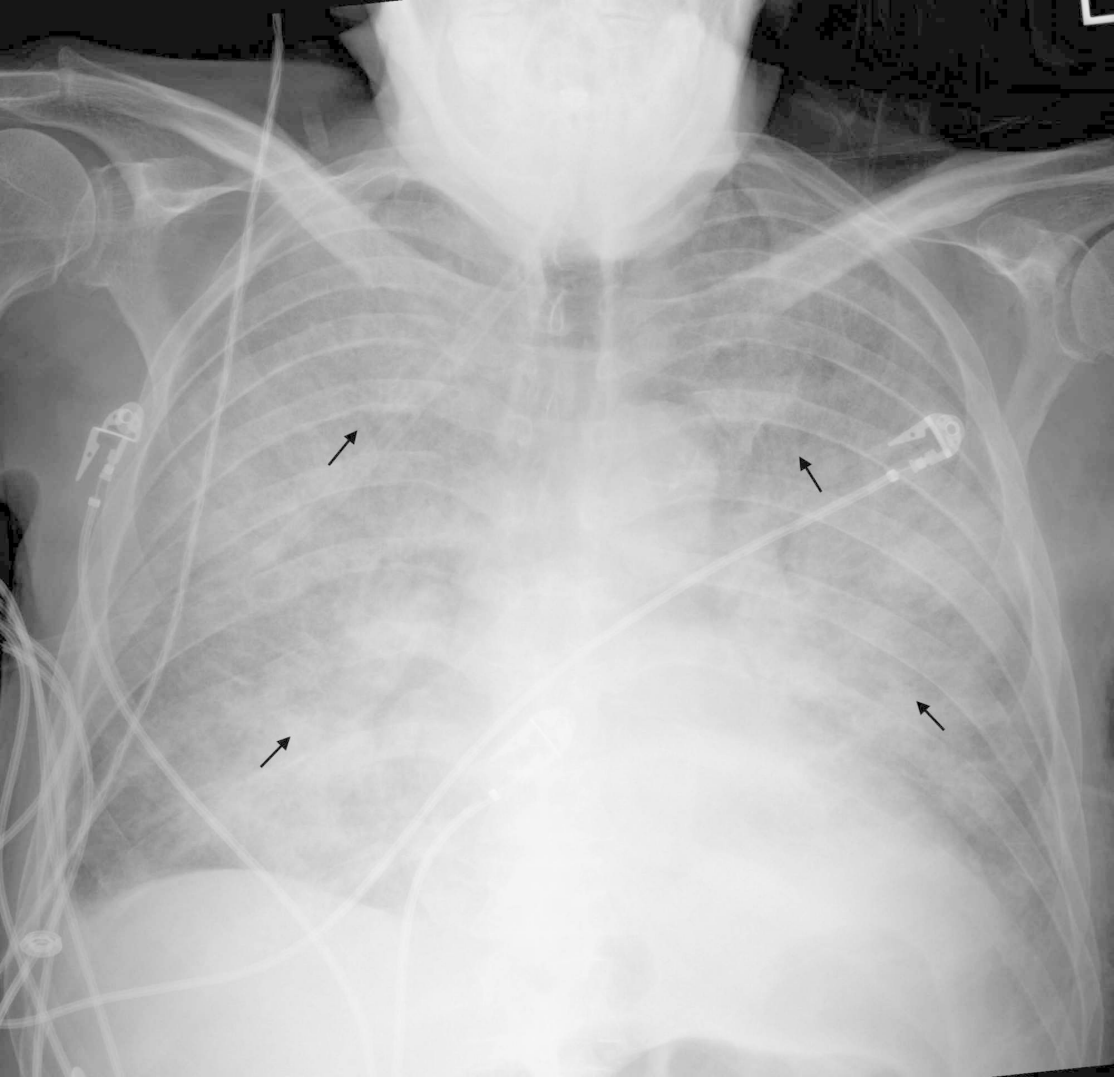
Portable anteroposterior chest radiography. Interval progression with development of extensive bilateral alveolar opacities (black arrows).

The patient became hypoxic overnight, and chest radiography revealed interval progression with the development of florid pulmonary edema and extensive alveolar opacities. The electrocardiogram did not reveal new ST-T changes. Serial troponin tests were negative. Bilevel positive oxygen therapy was started, and 100 mg of intravenous furosemide was administered over 12 hours. The patient’s oxygenation improved by the end of the night (Figure 2).

A fourth-generation HIV test was positive, the CD4 count was 50 cells/µL, and the HIV viral load was 7,078,332 copies/mL. Three sputum samples tested negative for acid-fast bacilli by Ziehl-Neelsen staining and an Xpert MTB\RIF assay (Cepheid, Sunnyvale, CA, USA).

**Figure 3:**
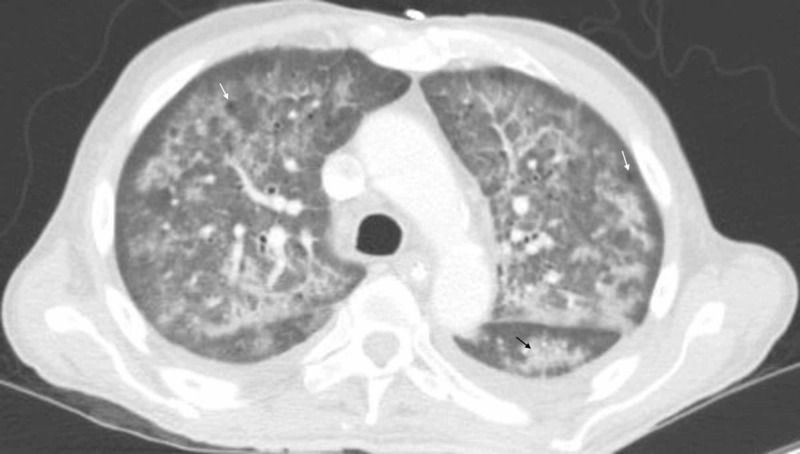
Contrast-enhanced CT of chest. Bilateral alveolar opacities ( black arrow ), pneumatoceles ( white arrows ), and bilateral small pleural effusions.

We examined the chest radiography results through the lens of HIV. PCP is the most common opportunistic infection in HIV patients and is a diagnosis that should not be missed in an AIDS patient with respiratory symptoms and a CD4 count of 50 cells/µL. Typical radiographic features include bilateral perihilar interstitial infiltrates, but chest radiographs are normal in 33% of cases [11]. Less common findings include solitary or multiple nodules, upper lobe infiltrates in patients receiving pentamidine, pneumatoceles, and pneumothorax [11]. Fever may be absent in 20% of cases. Cough is usually dry but can be productive in 30% of cases, and shortness of breath occurs in 68% of cases [12]. The duration of symptoms extends over weeks to months. Chest CT revealed bilateral alveolar opacities, pneumatoceles, and small bilateral pleural effusion (Figure 3).

We initiated empiric treatment with intravenous trimethoprim-sulfamethoxazole. Corticosteroid therapy was not indicated based on the severity of the patient’s illness. Urgent bronchoscopy and bronchoalveolar lavage (BAL) examination were performed and revealed PCP by Giemsa staining.

Our patient tolerated his treatment well and returned to living with his family in the Southeast United States. The TB sputum culture remained negative. We were last updated by the patient’s family five months after his discharge that he had started antiretroviral therapy, was attending an outpatient rehabilitation center once a week, and was planning to visit his home country in Africa in several weeks.

## Discussion

The patient in this case is one of the 1.1 million people in the USA who are living with HIV. In 2006, the Centers for Disease Control (CDC) recommended that adolescents and adults between the ages of 13 and 64 years should be screened for HIV infection and that the written informed consent requirement should be eliminated [13]. Our patient would not have been screened under the CDC guidelines. The state in which our patient lived did not eliminate the requirement for written informed consent for HIV testing until 2015 [14]. A routine opt-out HIV testing program in emergency departments would have probably captured his diagnosis during one of his several hospitalizations or ER visits if it had been in effect where he had received his medical care. Patients with limited English proficiency (LEP), such as our patient, have worse clinical outcomes than those with high proficiency and experience a higher incidence of medical errors resulting in physical harm [6, 15]. One study found that professional interpreters are often not used for patients with LEP: only approximately 60% of patients reported the presence of any kind of interpreter with physicians during their hospitalizations [16].

Diagnostic parsimony is a concept that physicians commonly utilize in their clinical reasoning. As physicians, we have correctly been taught to first look for a single unifying diagnosis that explains different symptoms, signs, and test results in our patients rather than to look for two or three unrelated diagnoses [17-18]. The concept of diagnostic parsimony is based on an ancient Greek philosophical principle that is summarized by the Latin phrase “entia non sunt multiplicanda praeter necessitate,” which translates to “entities must not be multiplied beyond necessity.” This principle is commonly known as Occam’s razor, named for the 14th-century philosopher William of Ockham [18-19]. Heart failure was the simplest and most likely diagnosis for our patient, especially because the patient also had a known history of heart failure and had been hospitalized several times for the condition.

Fortunately, we made the judgement that it was best not to rely on this principle in our search for a diagnosis in this case. We instead applied Hickam’s dictum, “patients can have as many diseases as they damn well please”, named after John Hickam, the Chair of Medicine at Indiana University (1914-1970). Ultimately, we arrived at three diagnoses: congestive heart failure, AIDS, and PCP [18-19]. The concept of diagnostic parsimony is not appropriate for immunocompromised patients, polypharmacy cases, and elderly patients, in whom disease presentations are atypical and having more than one diagnosis is not uncommon [17-18]. Diagnostic parsimony must be used with caution, and even arriving at an established diagnosis does not exclude the coexistence of others [20].

## Conclusions

HIV often masquerades as other illnesses and should therefore be considered and screened for in clinical practice. Clinicians must be skeptical; they should not fall into the trap of diagnostic momentum or accept diagnostic labels at face value. Our healthcare system failed our patient for several years; however, thinking outside the box enabled us to make the correct diagnosis in only a few days.
